# Neural Stem Cell Regulation by Adhesion Molecules Within the Subependymal Niche

**DOI:** 10.3389/fcell.2019.00102

**Published:** 2019-06-12

**Authors:** Jose Manuel Morante-Redolat, Eva Porlan

**Affiliations:** ^1^Departamento de Biología Celular, Biología Funcional y Antropología Física, Universitat de València, Burjassot, Spain; ^2^Centro de Investigación Biomédica en Red de Enfermedades Neurodegenerativas, Instituto de Salud Carlos III (ISCIII), Madrid, Spain; ^3^Estructura de Recerca Interdisciplinar en Biotecnologia i Biomedicina, Universitat de València, Burjassot, Spain; ^4^Departamento de Neuropatología Molecular, Centro de Biología Molecular Severo Ochoa Consejo Superior de Investigaciones Científicas – Universidad Autónoma de Madrid (CSIC-UAM), Madrid, Spain; ^5^Departamento de Biología Molecular, Facultad de Ciencias, Universidad Autónoma de Madrid, Madrid, Spain; ^6^Hospital La Paz Institute for Health Research (IdiPAZ), Instituto de Salud Carlos III (ISCIII), Madrid, Spain

**Keywords:** subependymal zone, adhesion molecules, extracellular matrix, neural stem cell, quiescence, niche, adult neurogenesis

## Abstract

In the mammalian adult brain, neural stem cells persist in neurogenic niches. The subependymal zone is the most prolific neurogenic niche in adult rodents, where residing stem cells generate large numbers of immature neurons that migrate into the olfactory bulb, where they differentiate into different types of interneurons. Subependymal neural stem cells derive from embryonic radial glia and retain some of their features like apico-basal polarity, with apical processes piercing the ependymal layer, and a basal process contacting blood vessels, constituting an epithelial niche. Conservation of the cytoarchitecture of the niche is of crucial importance for the maintenance of stem cells and for their neurogenic potential. In this minireview we will focus on extracellular matrix and adhesion molecules in the adult subependymal zone, showing their involvement not only as structural elements sustaining the niche architecture and topology, but also in the maintenance of stemness and regulation of the quiescence-proliferation balance.

## The Neurogenic Adult Subependymal Zone: A Polarized Epithelial Niche

In most adult tissues, stem cells dwell in specialized microenvironments called “niches” (Scadden, 2006). In the mammalian adult brain, the subependymal zone (SEZ), is a specialized niche that encompasses a thin layer of cells apposed to the ventricular wall, lined by ependymal cells (EC). Here, neural stem cells (NSC) coexist with their own progeny, supporting niche cells, neighboring blood vessels (BV), and a specialized extracellular matrix (ECM). In addition, they are in contact with the cerebrospinal fluid through a primary cilium at the end of a cytoplasmic process that traverses the ependymal layer through the center of a rosette of ependymocytes, named “pinwheel” (Mirzadeh et al., 2008; Lin and Iacovitti, 2015).

Residing NSC generate large numbers of immature neurons that migrate into the olfactory bulb (OB), to differentiate into several types of interneurons that contribute to refine the processing of olfactory information (Lim and Alvarez-Buylla, 2014; Merkle et al., 2014). The ceaseless production of neurons follows a hierarchical lineage where NSC divide to generate transit amplifying progenitors (TAP) and/or to self-renew. TAP cells can divide a few times more to expand future neural progeny to finally generate migrating neuroblasts (Ortega et al., 2013; Ponti et al., 2013). SEZ NSC also contribute to gliogenesis, however, much less productively than to neurogenesis (Menn et al., 2006; Ortega et al., 2013; Sohn et al., 2015), at least in homeostatic conditions.

**FIGURE 1:**
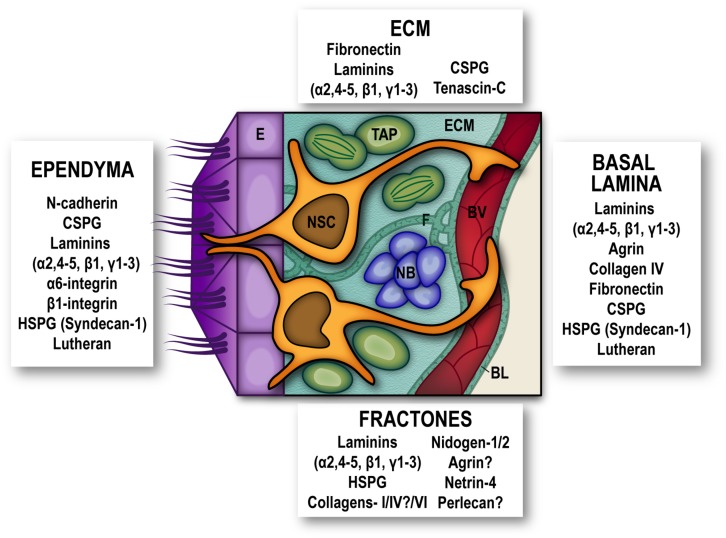
Extracellular matrix and adhesion molecules are SEZ niche components. Schematic representation of the subependymal niche showing cellular populations (multiciliated ependymal cells –E, purple-, neural stem cells –NSC, orange-, transit amplifying progenitors –TAP, green-, neuroblasts –NB, blue- and blood vessels –BV, red-) and specific extracellular matrix proteins and adhesion molecules reported to be present subependymal zone extracellular matrix (ECM), in basal lamina (BL), fractones (F), and ependymal cells. Question marks are present where conflicting reports have been published. For the sake of clarity other niche elements such as microglia, non-neurogenic astrocytes and innervation have been omitted from the schematic. HSPG, heparan-sulfate proteoglycans; CSPG, chondroitin sulfate proteoglycans.

In early developmental stages, ectodermally derived neural progenitors organize in a neuroepithelial layer surrounding the ventricles, termed the ventricular zone (VZ). Within this polarized arrangement, neuroepithelial cells (and later radial glial cells, RGC), exhibit a characteristic bipolar radial morphology supported by two points of adhesion: at their apical end, neighboring cells adhere to the luminal surface of the VZ through adherens junctions (AJs) mediated mainly by N-cadherin, whereas they remain attached to the subpial ECM *via* integrin–laminin interactions through their basal end-feet (Meng and Takeichi, 2009; Hirano and Takeichi, 2012). Embryonically, NSC derive from a pool of RGC that become specified at mid-gestation (Tramontin et al., 2003; Merkle et al., 2004; Kriegstein and Alvarez-Buylla, 2009; Fuentealba et al., 2015; Furutachi et al., 2015), and are as well glial in nature, displaying several ultrastructural characteristics of astrocytes (Doetsch, 2003). Additionally, they retain the apico-basal polarity reminiscent of RGC, with apical processes piercing the ependymal layer, and a basal process extruding onto the traversing BV and contacting their basal lamina (BL). This cytoarchitecture, with NSC spanning these two important signaling compartments categorizes the SEZ as an epithelial niche (Mirzadeh et al., 2008; Shen et al., 2008; Kokovay et al., 2010) (Figure 1).

## The ECM as a Niche Component

In most cellular contexts, cell-ECM adhesion mediated by receptors such as integrins provides not only physical support and positioning to the cells, but also initiates cellular responses mainly mediated by phosphorylation states and activities of cytosolic tyrosine kinases, which next regulate other kinases and scaffolding proteins to transduce signals (Morgan et al., 2007). By providing bidirectional connections (intracellularly, by the assembly of cytoskeleton and signaling complexes, and extracellularly, through interactions with ECM elements, and in some cases with counter-receptors on adjacent cell surfaces) integrins force spatial restrictions on signaling and ECM assembly, and so integrate cells with their microenvironment (Humphries et al., 2006). The adult SEZ is rich in ECM molecules, such as fibronectin, laminins-β1 and γ1, and chondroitin sulfate proteoglycans (Mercier et al., 2002; Marthiens et al., 2010) along with a layer of Tenascin-C that separates the SEZ from the adjacent striatum (Kazanis et al., 2007). Intriguingly, notwithstanding a relevance in embryonic and early postnatal NSC and neural progenitor proliferation and migration (Garcion et al., 2001), in the SEZ Tenascin-C deficiency does not affect NSC nor progeny (Kazanis et al., 2007).

Additionally, a specific feature of the SEZ ECM is the presence of “fractones,” conspicuous ECM structures thought to be merely extended formations of the vascular BL, which now seem to be functionally and structurally independent, with their own relevance in the SEZ niche. They may appear either as thin branching lines (stems) or as round deposits (bulbs) frequently popping out at the center of pinwheels or scattered along the inner ependymal wall, and associated with GFAP^+^-NSC (Nascimento et al., 2018; Sato et al., 2019). SEZ fractones contain laminins, N-sulfate heparan sulphate proteoglycans (HSPG), collagens-I/IV/VI, nidogen-1/2, agrin, netrin-4, and perlecan-1 (Mercier et al., 2002; Kerever et al., 2007; Douet et al., 2012; Mercier and Douet, 2014; Sato et al., 2019), and their source has been described as either ependymal (Nascimento et al., 2018) or originating from NSC themselves (Sato et al., 2019). Elimination of laminin-α5 from EC increased activation of NSC (Nascimento et al., 2018) and disrupting integrin-binding activities of laminins specifically in astrocytes (including NSC), decreased the number and size of fractones, although the effects of this disruption on NSC proliferation were not investigated *in vivo* (Sato et al., 2019). Interestingly, fractones can promote heparin-binding growth factor activity and influence cell proliferation in the SEZ by sequestering basic-FGF and BMP-4/7 from the extracellular milleu (Kerever et al., 2007; Douet et al., 2012; Mercier and Douet, 2014).

## Adhesion at the Basal End: Where NSC Meet the Blood Vessels and Their Basal Lamina

Around their basal process, RGC secrete a layer of ECM, to form the basement membrane, and transcriptome analyses of fetal human and embryonic mouse VZ, sub-VZ, and cortical plates, revealed elevated expression of genes related to cell adhesion and cell–ECM interactions (collagens, laminins, proteoglycans, and integrins) indicating their functional relevance (Fietz et al., 2012). Deletion of integrin function during brain development promotes process detachment, apoptosis, and altered neurogenesis (Graus-Porta et al., 2001; Campos et al., 2004; Belvindrah et al., 2007; Shen et al., 2008; Loulier et al., 2009; Fietz et al., 2010; Kazanis et al., 2010; Marthiens et al., 2010; Theocharidis et al., 2014; Chou et al., 2018), revealing altogether a structural role of integrin signaling within the niche, and in the maintenance of polarity, regulation of embryonic NSC pools asymmetric cell division, cortical expansion and neurogenesis.

Adult NSC are also polarized, and distinct stem-cell domains have been defined along their radial morphology. The basal domain corresponds to specialized long processes that contact directly BV, and the majority of dividing cells in the SEZ proliferate in the immediacy of BV which, ensheathed by their laminin-rich BL, provide the vascular compartment of the niche (Shen et al., 2008; Tavazoie et al., 2008). In fact, dividing NSC (and TAP) are directly associated with vasculature, establishing cell-ECM contacts with the endothelial BL (Shen et al., 2008). SEZ cells express differential levels of ECM receptors which appear to correlate with their mitotic status, rather than with a cell identity: for instance, quiescent NSC (qNSC) express low levels of α6β1-integrin, syndecan-1, and lutheran, whereas their levels increase in activated NSC (aNSC) and mitotic TAP (Shen et al., 2008; Kazanis et al., 2010; Morizur et al., 2018).

ECM *via* integrins regulates SEZ-cells proliferation and stemness, at least within this vascular context. Stromal-derived factor 1 (SDF1)- and CXC chemokine receptor 4 (CXCR4) upregulate EGFR and α6-integrin in aNSC and TAP, contributing to homing of SEZ progenitors to endothelial cells and to their proliferation by increasing their binding to laminin (Kokovay et al., 2010). *In vitro*, endothelial-derived laminin sustains proliferation and stemness of SEZ cells in brain endothelial-neurosphere cell co-cultures, process that is dependent on α6β1-integrin, *via* activation of the Notch and mTOR signaling pathways (Rosa et al., 2016). On the other hand, loss of integrin-linked kinase (ILK), enhances proliferation by over-activation of JNK (Porcheri et al., 2014). β1-integrin signaling suppresses astrocytic differentiation (Pan et al., 2014), and the carbohydrate-binding protein Galectin-1 interacts with β1-integrin to regulate the number of neural progenitors as well as new migrating neurons. This has a relevant implication on regeneration, as these new-born neurons enhanced recovery from behavioral deficits resulting from brain damage (Sakaguchi et al., 2006, 2010; Ishibashi et al., 2007). Contrasting downstream signals can be elicited by specific integrin ligands; transforming growth factor beta 1 (TGFβ1), an anti-inflammatory cytokine, exerts pro-neurogenic effects on SEZ NSC regulating a set of genes involved on the integrin pathway (Radice et al., 2015), and β8-integrin promotes proliferation of SEZ cells and in migrating neuroblasts, potentially through TGFβ1 as well (Mobley et al., 2009; Mobley and McCarty, 2011).

Although not considered strictly speaking as “cell adhesion,” it is important to highlight that vascular endothelial cells can also provide NSCs with a pro-quiescence environment by means of direct cell-cell contacts of NSCs with BV, since it has been shown that endothelial ephrinB2 and Jagged suppress NSC proliferation while maintaining stemness (Ottone et al., 2014). Also, cell–cell interactions with other niche dweller populations work in a negative feedback loop to prevent NSC exhaustion. Niche-residing non-neurogenic astrocytes, for instance, secrete delta-like homolog (Dlk1) that binds its own membrane-bound isoform expressed in the surface of NSC to regulate their self-renewal (Ferron et al., 2011). Additionally, NSCs and TAPs express surface-bound Notch-ligand Dll1 to sustain quiescence in qNSC that specifically express Notch2 receptor (Kawaguchi et al., 2013; Llorens-Bobadilla et al., 2015).

## Adhesion at the Apical End: Reaching the Ependymal Wall and Beyond

Distance from the EC/ventricle also seems to have a strong role limiting proliferation (Kazanis et al., 2010), indicating that elements involved in the maintenance of topology have functional roles in the SEZ neurogenic activity. According to their conserved apico-basal morphology, NSC directly contact the ependymal layer, with apical processes serving as grips that provide structural integrity. At the tip of the apical processes, a primary cilium pokes out from the ventricle wall serving as an antenna for the NSC (Mirzadeh et al., 2008).

Embryonic RGC bipolar radial morphology is supported at their apical tip through N-cadherin mediated AJs, to attach to the luminal surface of the VZ and neighboring cells (Hirano and Takeichi, 2012; Miyamoto et al., 2015). Compromised expression of N-cadherin in embryonic NSC leads to VZ/sub-VZ disruption, displacement of NSCs into the CSF, hydrocephalus, atypical neurogenesis and randomization of the intra-cortical structures and formation of periventricular heterotopias (Kadowaki et al., 2007; Gil-Sanz et al., 2014; Guerra et al., 2015; Jossin et al., 2017).

Attempts to inactivate N-cadherin specifically in postnatal/adult NSC have been made and, as in the embryo, N-cadherin-mediated adhesion is paramount to preserve the integrity of the adult SEZ and essential for the maintenance of NSC. hGFAP-Cre mice transgenic strain was used to evaluate the effect of N-cadherin in NSC from the SEZ. However, since both adult NSC and ependymocytes derive from RGC, which activate this promoter during development, unsurprisingly, both populations appeared drastically affected. The mutant displayed severe disassembly of the ependymal barrier concomitant with SEZ hyperplasia and increased proliferation of NSC, revealing that N-cadherin anchors act as quiescence signals (Porlan et al., 2014). In adult mice, acute inactivation of N-cadherin either in the whole SEZ or only in ECs showed an increase in NSC proliferation. In the case of ependymal inactivation, an additional denudation of the ventricle wall sheathing, in line with a previous report in which electroporation of a dominant-negative version of N-cadherin provoked ependymal loss and protrusion of the SEZ cells into the ventricle (Barnabe-Heider et al., 2008). These experiments clearly indicated that N-cadherin does indeed maintain the cytoarchitecture of the adult neurogenic niche and that this is functionally related to the activation status of residing stem cells (Porlan et al., 2014). E-cadherin, on the other hand, is the main component of the *zonula adherens* in non-neural epithelia, but maybe due to its secondary role in neural tissue, deletion of E-cadherin in the adult SEZ did not result in severe disruption of its cytoarchitecture, although still caused defects on NSC self-renewal (Karpowicz et al., 2009). Likewise, the lateral membrane adaptor protein Ank3 is critical for differentiation of EC and consequently, for neurogenesis (Paez-Gonzalez et al., 2011).

Integrins also contribute to the attachment of apical processes in embryonic RGC (Lathia et al., 2007; Loulier et al., 2009). In the adult SEZ, β1-integrin upregulates in NSC that activate to repopulate the niche after a depletion paradigm with antimitotic drugs. Also, injection of function-blocking antibodies in the adult ventricle in homeostasis results in disruption of the ventricular surface, increased TAP proliferation and invasion of neuroblast clusters within the ventricle, though NSC remained unaffected (Kazanis et al., 2010).

Preservation of quiescence and transition into an active proliferating state is an extremely regulated process. Therefore, is not surprising to find that extrinsic and intrinsic factors fine-tune the adhesive properties of the niche to retain stem cells and regulate their activation, adding another layer of complexity to the microenvironment. For example, N-cadherin dependent activation of NSC can be modulated by the proteolytic activity of specific proteases, such as Mmp24-MT5 that cleaves N-cadherin to properly activate NSC under physiological and regenerative conditions (Porlan et al., 2014). Furthermore, in experimental demyelination, A Disintegrin and metalloproteinase domain-containing protein 10 (ADAM10) processes N-cadherin in response to an activation signal initiated by EGFR (Klingener et al., 2014) again supporting the idea that dynamical regulation of adhesion to the niche by proteolysis can recruit NSC on demand for active proliferation. Interestingly, MMP12 another matrix metalloproteinase, regulates EC maturation and SEZ output modulating NSC quiescence (Shan et al., 2018).

Vascular Cell Adhesion Molecule-1 (VCAM1) is also highly expressed by NSC, specifically at their end-feet in the center of pinwheels to maintain NSC quiescence. Blocking VCAM1 function severely disrupts the niche structure, affecting ependymal cytoarchitecture and generating loss of pinwheels. This also produces an increase of OB neurogenesis, further showing that exogenous manipulation of the adhesive properties of the niche could indeed have an impact of neuronal progeny (Kokovay et al., 2012). Interestingly, VCAM1 increases as a response to IL-1β, signaling *via* NOX2-produced reactive oxygen species, to maintain NSC indicating that it can sense the environment, responding to chemokines involved in tissue repair (Kokovay et al., 2012).

## Adhesion and Regulation of Quiescence by the Niche

Notwithstanding their potential for generating differentiated progeny, NSC display a functional quiescence in adulthood (Fuentealba et al., 2015; Furutachi et al., 2015), and in contrast to other tissues, adult NSC can be found at different states of activation (Daynac et al., 2013; Codega et al., 2014; Mich et al., 2014; Llorens-Bobadilla et al., 2015; Chaker et al., 2016; Kalamakis et al., 2019). Switch from qNSC to aNSC state can be induced by selective elimination of neural progeny (Daynac et al., 2013; Codega et al., 2014; Mich et al., 2014), and quiescence appears to be a mechanism to protect NSC pools throughout life and hence maintain homeostasis and tissue regeneration. Failure to restrict mitotic activation of NSC leads to premature depletion of the niche (Doetsch et al., 2002; Molofsky et al., 2003, 2005, 2006; Kippin et al., 2005; Marques-Torrejon et al., 2013; Porlan et al., 2013), and in the aged brain, NSC quiescence increases in what has been interpreted as a mechanism to avoid full exhaustion (Luo et al., 2006; Bouab et al., 2011; Silva-Vargas et al., 2016; Bast et al., 2018; Kalamakis et al., 2019). In light of all these evidences, the quiescent state of NSC is now considered as an actively regulated condition, in contrast to the classic vision of it being a mere passive quality. Interestingly, the niche appears to play an essential role in the regulation of NSC fate and number by controlling the reversible transition between the quiescent and active NSC compartments (Llorens-Bobadilla and Martin-Villalba, 2017; Basak et al., 2018; Kalamakis et al., 2019).

**FIGURE 2:**
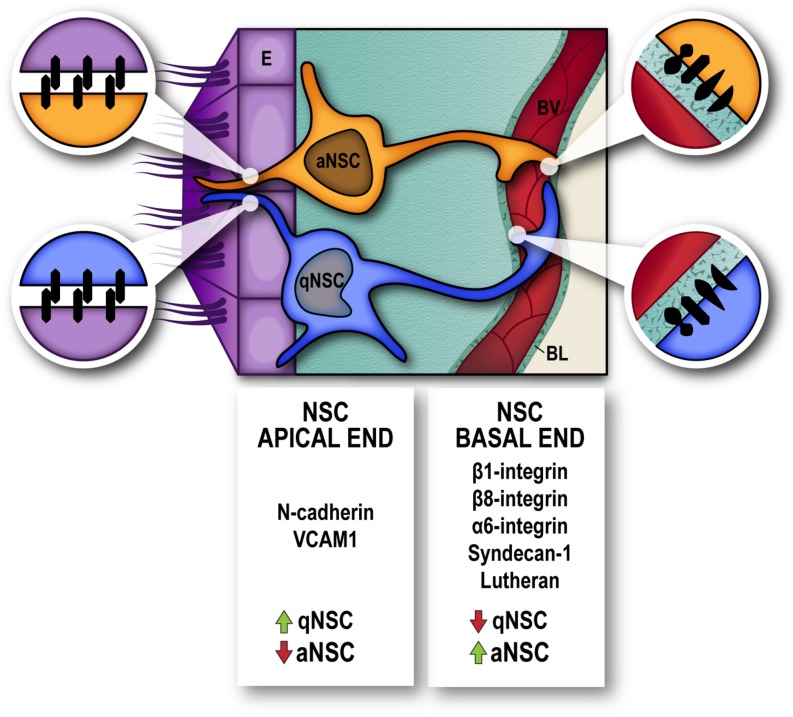
Molecules involved in cell-cell adhesion and interaction with the niche extracellular matrix, actively participate in the regulation of neural stem cell dormancy/activation. Cell-cell junctions between Neural Stem Cells (NSC) and Ependyma (E) at the apical end (magnified circles, left) and Cell–Extracellular matrix interactions between NSC and endothelial basal lamina (BL) at the basal end (magnified circles, right), in the vicinity of blood vessels (BV), regulate the quiescence-activation state of NSC. High levels of N-cadherin and VCAM1 serve to maintain position and dormancy of quiescent neural stem cells (qNSC) within the niche by their apical end, whilst integrins α6/β1 and β8 and syndecan-1 and lutheran receptors are upregulated in neural stem cells that are actively proliferating (aNSC).

Many efforts have been made to reveal the molecular signature of qNSC, and strategies to prospectively isolate dormant NSC from the adult SEZ have used a combination of stem cell/progeny markers to analyze the transcriptome of non-proliferative vs. aNCS (Capela and Temple, 2002; Pastrana et al., 2009; Beckervordersandforth et al., 2010; Daynac et al., 2013; Codega et al., 2014; Mich et al., 2014; Llorens-Bobadilla et al., 2015; Chaker et al., 2016; Morizur et al., 2018). Transcriptome analyses have helped to disclose the integration of signals from the microenvironment that actively maintain the quiescent state. Not surprisingly, a very significant contribution of molecules involved in cell adhesion and interaction with the niche milleu have been found to actively maintain dormancy. Amongst the most represented GO categories in qNSC are cell–cell adhesion, ECM-response and anchorage-dependent niche signals, cell communication, and signaling receptors. Most genes enriched in qNSC encoded membrane-associated proteins, underscoring the key role played by the microenvironment in the regulation of the quiescent state in the adult SEZ. Of relevance, VCAM1 and N-cadherin previously reported as regulators of NSC quiescence (Kokovay et al., 2012; Porlan et al., 2014) were specifically found increased in prospectively isolated qNSC, as well as other cadherins, protocadherins, neural cell adhesion molecule 1/2 and ECM-components, whereas syndecan-1 was overexpressed specifically in aNSC (Morizur et al., 2018) as previously described (Kazanis et al., 2010). Interestingly, syndecans play their functions as cell surface receptors by acting as both adhesion and docking receptors, and thus are capable of regulating both intra- and extracellular activities and can recruit soluble growth factors, matrix metalloproteinases, chemokines and cytokines to the cell surface (Kwon et al., 2012) (Figure 2).

## Concluding Remarks

Within the SEZ niche ecosystem, NSC necessarily communicate with their neighboring cellular populations and surrounding matrix, and such interaction is essential for the maintenance of stem cell identity and control of the timing and mode of cell division. Indeed, cell-to-cell contacts and cell-ECM adhesion not only provide tissue integrity, cell orientation and topology, but actively foster NSC self-renewal and maintenance by either placing stem cells in proximity of different signaling sources, or directly participating in the signaling process, since most adhesion molecules act as receptors and signal transductors. Many of the signaling cues that maintain NSC positioning, and some of the molecular mechanisms that trigger the switch from dormancy toward proliferation in physiological and pathological conditions to promote tissue regeneration, are starting to emerge. Amongst these, ECM and cell adhesion molecules play a crucial role, highlighting that the niche allows a relative plasticity whose manipulation provides an important window for regeneration.

## Author Contributions

EP conceived the manuscript. EP and JMM-R wrote the manuscript.

## Conflict of Interest Statement

The authors declare that the research was conducted in the absence of any commercial or financial relationships that could be construed as a potential conflict of interest.
